# Study on Synthesis and Performance of a Hybrid Crosslinked Composite Gel for High-Temperature Lost Circulation Control

**DOI:** 10.3390/gels12040325

**Published:** 2026-04-11

**Authors:** Jiangang Shi, Xuyang Yao, Chaofei Wang, Tao Ren, Kecheng Liu, Huijun Hao, Zhangkun Ren, Jingbin Yang

**Affiliations:** 1 Oil Production Technology Research Institute of PetroChina Xinjiang Oilfield Company, Karamay 834000, China; 2 CNPC Engineering Technology R&D Company Limited, Beijing 102206, China; 3 School of Petroleum Engineering, China University of Petroleum (East China), Qingdao 266580, China

**Keywords:** gel material, drilling fluid, lost circulation control, hybrid crosslinking, rheological properties

## Abstract

Conventional chemical gel plugging materials often suffer from poor high-temperature stability and inadequate mechanical properties. To address these issues, this study developed a high-performance composite gel material using a multi-component hybrid crosslinking strategy. The material employs γ-methacryloxypropyltrimethoxysilane (MPTMS) as the silica source, which hydrolyzes in situ to generate SiO_2_, thereby enhancing temperature resistance. Laponite nanoplatelets are incorporated as a toughening agent and physical crosslinking points, while a self-synthesized reactive microgel (BWL) serves as the organic crosslinking core. Through copolymerization with monomers such as acrylamide (AM) and methacrylic acid (MAA), a triple-crosslinked network structure is constructed. Compared with conventional gels, the synthesized hybrid crosslinked composite gel maintains a high storage modulus and loss modulus after aging at 140 °C and exhibits excellent tensile and compressive properties. Furthermore, the gel was processed into particle-based lost circulation materials with different particle sizes. High-temperature and high-pressure plugging experiments demonstrate that when using a mixed system of 40–60 mesh, 20–40 mesh, and 10–20 mesh gel particles with a total concentration of 2%, it can effectively seal highly permeable sand beds and fractures with apertures up to 5 mm. This meets the engineering requirements for lost circulation materials with high strength and high stability in deep, high-temperature formations.

## 1. Introduction

Lost circulation is one of the most severe downhole complications in drilling engineering. It not only directly causes drilling fluid loss, time delays, and cost increases but can also lead to secondary disasters such as wellbore instability, stuck pipe, and even blowout if improperly handled. These pose serious threats to the safety and efficiency of oil and gas exploration and development [1,2,3]. As exploration extends into deep, ultra-deep, and complex geological areas, formation temperatures and pressures continue to rise. Fractures and pores are more developed [4,5,6,7]. This places higher demands on the efficiency and reliability of lost circulation materials (LCMs).

Chemical gel-based LCMs show significant potential for tackling complex loss formations. This is due to their unique viscoelastic deformation and adaptive plugging capabilities. Unlike traditional rigid materials (e.g., calcium carbonate, walnut shells) or fibrous materials, gels possess good deformability, recovery, and interfacial adhesion. Driven by formation pressure, they can extrude into loss channels. Through an “adaptive filling–compaction–filming” process, they form a tight sealing layer. This enables effective plugging of various loss channels, from micro-fractures to millimeter-scale channels. They demonstrate good engineering applicability and broad-spectrum sealing performance [8,9,10,11]. However, conventional chemical gel systems, especially polyacrylamide-based ones, have notable performance shortcomings in high-temperature, high-pressure (HTHP) conditions. First, their thermal stability is insufficient. Under high temperatures, polymer chains are prone to hydrolytic scission, oxidative degradation, or premature crosslinker failure. This leads to the destruction of the gel network, a sharp decline in strength, or even phase separation/liquefaction [12]. Second, their mechanical properties are generally weak. They often exhibit low storage modulus, insufficient toughness, and poor resistance to compression and shear. Under downhole high confining pressure and dynamic fluid erosion, they are prone to structural collapse, extrusion, or localized rupture. Maintaining a long-term, effective pressure-bearing sealing barrier is difficult [13].

To improve the temperature resistance and mechanical properties of gels, researchers have introduced various reinforcing materials. Li et al. [14] developed a gel LCM resistant to 140 °C by combining partially hydrolyzed polyacrylamide with fibers. However, the interfacial compatibility and long-term thermal aging stability between the fibers and the matrix still need improvement. Nano-silica (SiO_2_) is a common reinforcing filler that can enhance polymer thermal stability and modulus [15]. However, Lin et al. [16] pointed out that untreated SiO_2_ tends to agglomerate in the matrix. It bonds with polymer chains only through weak physical interactions, resulting in low stress transfer efficiency and limited toughness improvement. Laponite is another common nano-reinforcing material. Its disc-shaped nanoplatelets can exfoliate and disperse in water, acting as crosslinking points during polymerization. This forms tough, self-healable composite hydrogels [17,18,19]. However, Haraguchi et al. [20] showed that gels relying solely on Laponite physical crosslinking have limited rigidity enhancement. Their long-term structural stability under HTHP conditions remains inadequate. Therefore, constructing a composite reinforcing system that synergistically improves gel temperature resistance, rigidity, and toughness is key to developing LCMs suitable for deep, high-temperature formations. In recent years, organic–inorganic hybridization and multi-network design have provided new pathways for developing high-performance gels [21,22,23]. Chemically anchoring the inorganic reinforcing phase into the organic polymer network can significantly improve interfacial compatibility and achieve synergistic performance enhancement.

Previous studies have predominantly employed single reinforcement strategies or simple physical blending approaches, making it challenging to simultaneously achieve high-temperature resistance, rigidity, toughness, and long-term structural stability in gel materials. To address this challenge, we propose a triple-network hybrid crosslinking strategy in this work: using a reactive microgel (BWL) as the organic crosslinking core, in situ hydrolyzed SiO_2_ from MPTMS to enhance temperature resistance and rigidity, and Laponite nanoplatelets for physical toughening. Through copolymerization of AM and MAA, a hybrid gel material was synthesized that exhibits excellent viscoelasticity, high-temperature resistance, and superior mechanical properties. The synthesis principle was systematically elucidated, and the rheological properties, tensile strength, and compressive strength of the gel before and after high-temperature aging were thoroughly investigated, followed by evaluation of its plugging performance. In contrast to conventional polymer-silica composite gels, our approach employs MPTMS as a bifunctional coupling agent that hydrolyzes in situ to generate SiO_2_ covalently bonded to the polymer network. This eliminates interfacial debonding and enhances stress transfer efficiency. Similarly, while Laponite-reinforced hydrogels have been reported to exhibit improved toughness through physical crosslinking, their rigidity and long-term thermal stability remain limited. By combining Laponite with covalently anchored SiO_2_ and the high-density organic crosslinking network formed by BWL microgel, our triple-crosslinked system achieves synergistic enhancements in temperature resistance, rigidity, and toughness—a comprehensive performance profile that distinguishes it from previously reported single- or dual-network systems.

## 2. Results and Discussion

### 2.1. Synthesis Principle of the Hybrid Crosslinked Composite Gel

**Figure 1:**
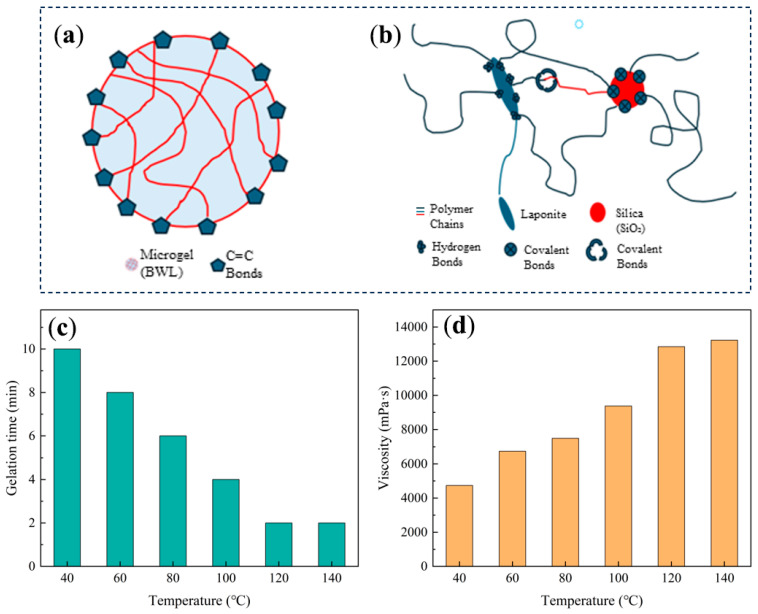
Schematic illustration of reactive microgel BWL and internal crosslinked structure of the gel: (**a**) Reactive microgel BWL. (**b**) Polymer chain/Laponite/SiO_2_ crosslinking structure. (**c**) Variation in gelation time with temperature. (**d**) Variation in gel viscosity with temperature.

A self-made reactive microgel BWL was used as the organic crosslinking agent during synthesis. The C=C bonds in organic crosslinking agents play a crucial role in monomer polymerization crosslinking. Compared with conventional organic crosslinking agents (such as N,N′-methylenebisacrylamide and N-hydroxymethyl acrylamide), the reactive microgel BWL features a high density of active C=C double bonds on its surface. When BWL undergoes crosslinking reactions with AM and MAA monomers, the resulting crosslinking density is significantly higher than that achieved using conventional organic crosslinking agents. This leads to the formation of a dense three-dimensional network structure. The synthesized gel exhibits excellent thermal stability, as shown in Figure 1a.

As a bifunctional compound, MPTMS contains a C=C double bond in its main chain, which can participate in polymerization reactions with organic components. Additionally, the methoxysilane groups in its side chains can interact physically or chemically with inorganic components [12]. Under the influence of MAA, MPTMS undergoes hydrolysis to generate silanol groups, which can self-polymerize to form SiO_2_, react with other polymers via silanol covalent bonds, or condense with the nanosheet layers on the Laponite surface. Simultaneously, the vinyl groups in MPTMS can copolymerize with AM and MAA monomers to form polymer chains [13], as shown in Figure 1b. Consequently, the SiO_2_ generated from MPTMS hydrolysis serves as an inorganic component chemically bonded to the organic matrix, thereby enhancing the compatibility and dispersibility of SiO_2_ within the polymer matrix. Laponite, acting as a toughening agent, embeds its layered structure into the polymer chains through non-covalent interactions. This reinforcement mechanism strengthens the spatial network structure while imparting flexibility to the polymer chains. Through the synergistic effects of organic crosslinking, covalent anchoring, and physical toughening, the three crosslinking components collectively construct a hybrid crosslinked composite gel characterized by a dense network structure, excellent mechanical properties, and good thermal stability. Furthermore, Figure 1c,d illustrate the effects of temperature on gelation time and viscosity, respectively. As the temperature increases, the gelation time gradually decreases. In the temperature range of 40–120 °C, the gelation time decreases significantly; beyond 120 °C, the decreasing trend becomes more gradual. Meanwhile, gel viscosity initially increases and then tends to plateau with increasing temperature. At 140 °C, the viscosity reaches 13,227 mPa·s, with a corresponding gelation time of 2 h. This behavior can be attributed to the influence of temperature on the hydrolysis and condensation reaction kinetics as well as molecular mobility. Moderate temperature elevation accelerates the hydrolysis and condensation of MPTMS, promoting rapid formation of the crosslinked network and thereby shortening the gelation time. However, when the temperature becomes excessively high, the reaction rate approaches saturation, resulting in less pronounced changes in gelation time, while the gel network structure becomes fully developed and the viscosity stabilizes. Compared with the conventional gel systems reported in previous studies [14,15,16], the triple-network hybrid crosslinked composite gel developed in this study exhibits clear advantages in terms of thermal stability.

**Figure 2:**
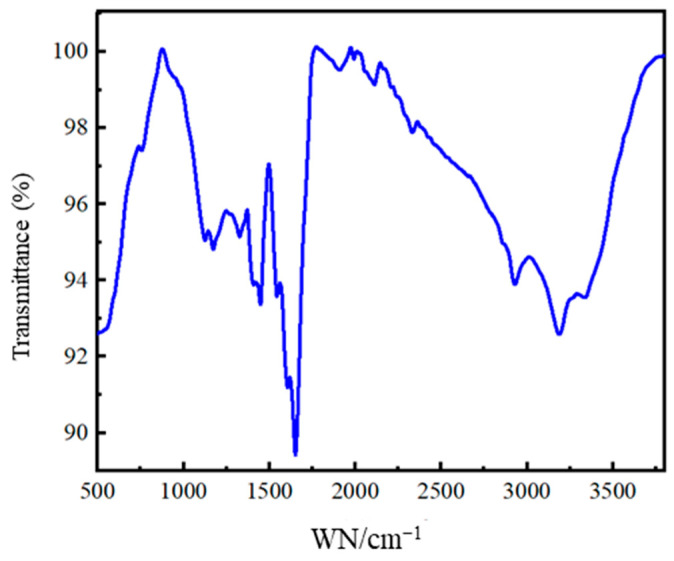
FTIR spectrum of the hybrid crosslinked composite gel.

Figure 2 presents the Fourier transform infrared (FTIR) spectrum of the hybrid crosslinked composite gel. As shown in Figure 2, the absorption peak observed near 1172 cm^−1^ is attributed to the antisymmetric stretching vibration of Si–O–Si bonds, while the peaks around 416 cm^−1^ and 480 cm^−1^ correspond to the bending vibrations of Si–O bonds, indicating the successful formation of Si–O–Si structures within the gel [13]. Compared with the strong and broad absorption band of nano-SiO_2_ powder centered around 1100 cm^−1^, the corresponding peak in the gel is notably weaker in intensity, suggesting that SiO_2_ is not simply dispersed as a physical filler within the gel. Combined with the presence of Si–O bond vibrations, this observation indicates that covalent interactions occurred between SiO_2_ and Laponite in the gel. Furthermore, the characteristic absorption peaks of C=C bonds from the reactive microgel BWL and MPTMS molecules are not clearly observed in the FTIR spectrum, confirming that BWL underwent copolymerization with MPTMS, AM, and MAA [13].

Based on the above analysis, unlike conventional chemically crosslinked gels, the synthesized hybrid crosslinked composite gel features a triple crosslinking structure: (1) The reactive microgel BWL acts as an organic crosslinking agent, copolymerizing with AM, MAA, and MPTMS monomers to form a high-density polymer chain network. (2) With MAA as a silane hydrolysis catalyst and MPTMS as the silica source, hydrolysis produces silanol groups, a portion of which condense with each other to form Si–O–Si structures (SiO_2_ nanoparticles) that are embedded into the polymer network framework via covalent bonds. (3) Acting as a physical crosslinking agent, Laponite clay platelets interact with polymer chains through surface hydrogen bonding and can also undergo condensation with SiO_2_ particles to form covalent linkages.

**Figure 3:**
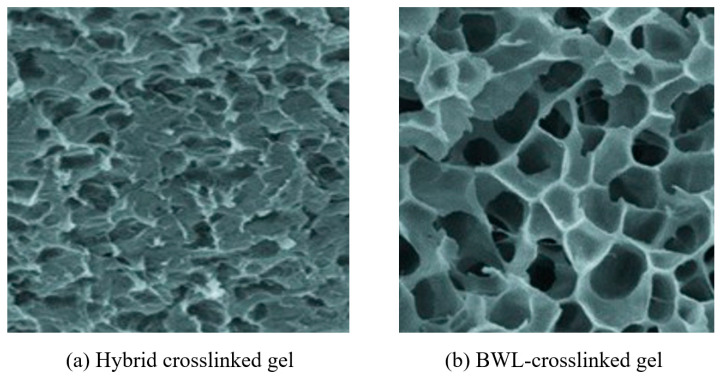
SEM micrographs of gels with different crosslinking characteristics.

Compared with conventional chemically crosslinked gels, the hybrid crosslinked composite gel synthesized on the basis of the above multi-network structural design integrates the good viscoelasticity of monomer-polymerized gels, the strong temperature resistance of SiO_2_-reinforced gels, and the excellent mechanical properties of Laponite-reinforced gels. After freeze-drying, the microstructure of the hybrid gel was examined by scanning electron microscopy (SEM) (Figure 3a). Overall, it exhibits a honeycomb-like porous morphology with pore diameters of approximately 200–1000 nm. As shown in Figure 3b, the gel prepared using BWL alone as the crosslinking agent with AM and MAA also presents a honeycomb morphology, but with a markedly higher pore quantity and larger pore diameters (>1 μm). Due to the triple crosslinking structure, the hybrid crosslinked composite gel attains a significantly higher crosslinking degree and a stiffer framework, thus exhibiting superior mechanical and temperature resistance performance.

**Figure 4:**
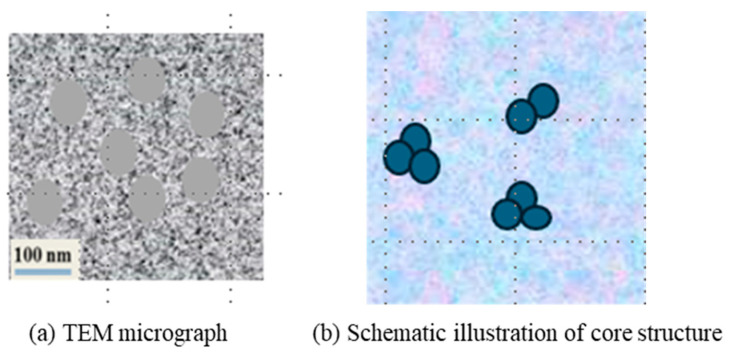
TEM micrograph and schematic illustration of the core structure in the hybrid crosslinked composite gel.

To investigate the internal structural composition of the hybrid crosslinked composite gel, transmission electron microscopy (TEM) characterization was performed on the prepared gel samples. The microstructure is shown in Figure 4a. The results indicate that there is an obvious “core” structure inside the gel, but the core size is not uniform, with a particle size range of 30–100 nm. Based on the gel composition and reaction mechanism, both Laponite and SiO_2_ derived from MPTMS hydrolysis can serve as potential core structures. Studies by Yang [24] and Chen [25] have shown that when the mass concentration of Laponite in aqueous solution is low (<1%), Laponite can form a uniform dispersion after sufficient stirring and hydration without forming obvious aggregation structures. The addition of AM and AMPS molecules will enhance the dispersion degree of Laponite. When the concentration of Laponite is high (>2%), the negatively charged surface and positively charged edge of Laponite particles will form a “card-house” aggregation structure through electrostatic attraction, which will gel the dispersion solution. Due to the large number of Laponite aggregation structures, the TEM microstructure of the gel solution will show a continuous aggregation morphology. Since the core structure in the TEM microstructure of the hybrid crosslinked composite gel is dispersed and the mass concentration of Laponite in the gel composition is only 0.8%, the core structure of the gel is not Laponite aggregates. Thus, the core structures in the hybrid gel are attributed to SiO_2_ nanoparticle aggregates formed via MPTMS hydrolysis. This indicates that SiO_2_ nanoparticles are closely connected to each other and embedded in the gel framework, thereby enhancing the structural strength and stability of the gel.

### 2.2. Rheological Properties of the Hybrid Crosslinked Composite Gel

The rheological properties (viscoelasticity, tensile resistance, and compression resistance) of the gel lost circulation material determine its applicable range. The variation in gel viscoelasticity with temperature reflects its temperature resistance performance, while its tensile and compression resistance indicate its stability under high-pressure conditions when subjected to compression and tension [26]. Given the high risk of well leakage in deep, high-temperature formations and the high-performance requirements for lost circulation materials, this study mainly investigates the effect of temperature on the viscoelasticity, tensile resistance, and compression resistance of the hybrid crosslinked composite gel. It is important to note that the gel samples used in these tests were prepared into specific shapes; their properties are identical to those of gel particles and thus can accurately represent the performance of the gel particles within lost circulation pathways.

**Figure 5:**
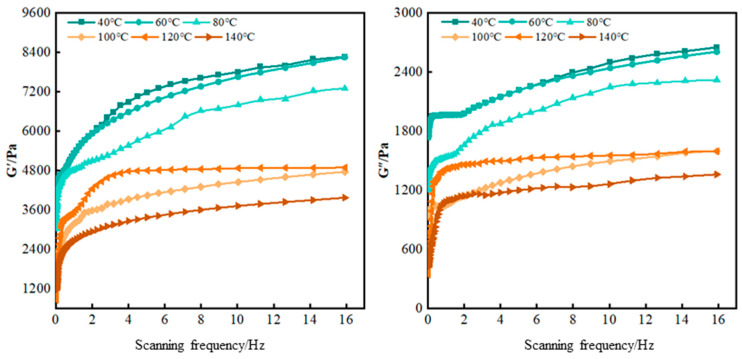
Variation in viscoelasticity of hybrid crosslinked composite gel under different temperature conditions.

The hybrid crosslinked composite gel was aged at temperatures ranging from 40 °C to 140 °C for 48 h, and its viscoelastic properties, tensile resistance, and compression resistance were tested. The storage modulus (G′) and loss modulus (G″) under different temperature conditions are shown in Figure 5.

As shown in Figure 5, within the scanning frequency range of 0–16 Hz, the storage modulus (G′) and loss modulus (G″) increase with frequency under various temperature conditions. The storage modulus consistently exceeds the loss modulus. When the scanning frequency ranges from 0 to 4 Hz, the storage modulus increases significantly; then, the growth slows down and eventually stabilizes at around 7800 Pa. When the scanning frequency is from 0 to 2 Hz, the loss modulus increases significantly and then grows slowly, with an overall increase smaller than that of the storage modulus. It eventually stabilizes at around 2500 Pa. Under the temperature range of 40 °C to 140 °C, both the storage modulus and loss modulus of the gel decrease as the temperature rises. Taking the scanning frequency of 16 Hz as an example, the storage modulus and loss modulus do not change significantly between 40 and 60 °C. As the temperature increases to 100 °C, the storage modulus decreases to around 4800 Pa, and the loss modulus also dramatically decreases to around 1500 Pa. When the temperature is between 100 and 120 °C, the storage modulus and loss modulus do not decrease significantly. As the temperature further increases to 140 °C, both the storage modulus and loss modulus show a decreasing trend again. According to the classification of gel viscoelasticity and practical experience in oilfield applications, when the storage modulus of the gel is higher than 4000 Pa, and the loss modulus is higher than 1000 Pa, it can meet the application requirements of lost circulation materials. Therefore, the hybrid crosslinked composite gel can be applied for lost circulation control in high-temperature formations up to at least 140 °C. This excellent viscoelasticity enables the gel to maintain structural integrity when entering loss channels, undergoing moderate deformation under pressure without fracturing, thereby achieving a plugging process characterized by “adaptive filling, compaction, and film formation.”

According to the analysis of the synthesis principle of the hybrid crosslinked composite gel, the addition of SiO_2_ generated by the hydrolysis of MPTMS and Laponite results in a triple crosslinked structure (BWL crosslinked structure, SiO_2_ crosslinked structure, and Laponite crosslinked structure) in the gel. At relatively lower temperatures, all three structures are less affected by temperature. As the temperature increases (60–100 °C), the molecular motion becomes more intense, and the hydrogen bonds formed between Laponite and the polymer molecular chains in the spatial structure begin to be destroyed due to the temperature effect. This results in a significant decrease in the storage modulus and loss modulus of the gel [27]. When the temperature continues to rise (>140 °C), the covalent bonds between SiO_2_, Laponite, and the polymer molecular chains are also affected by the temperature and begin to break. As a result, the viscoelasticity of the gel will further decline under high-temperature conditions.

**Figure 6:**
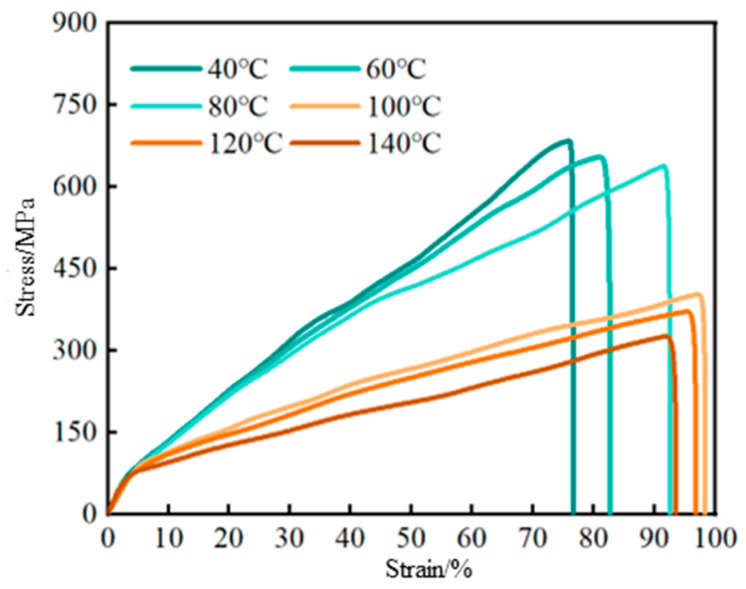
Variation in the tensile strength of hybrid crosslinked composite gel under different temperature conditions.

**Figure 7:**
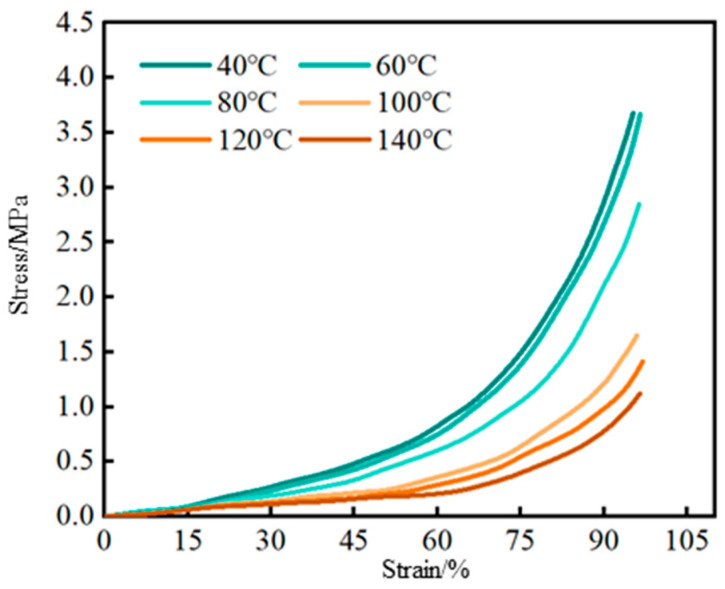
Variation in the compressive strength of hybrid crosslinked composite gel under different temperature conditions.

After gelation, the hybrid crosslinked composite gels were aged for 48 h at various temperatures. Their tensile and compressive strength test results are shown in Figure 6 and Figure 7, respectively. As shown in Figure 6, with increasing strain, the tensile stress on the gel rapidly increases until the gel sample fractures. The tensile strength of the gel shows little change between 40 °C and 60 °C, decreases significantly between 60 °C and 100 °C, and varies only slightly between 100 °C and 140 °C. This behavior is mainly attributed to the addition of SiO_2_ generated from the hydrolysis of MPTMS and Laponite, which form crosslinking points through hydrogen bonds and covalent bonds, thereby enhancing the gel’s tensile strength. The gel structure exhibits strong stability at 40–60 °C. Between 60 °C and 100 °C, the hydrogen bonding between polymer chains weakens, and molecular motion intensifies, leading to increased strain and a significant reduction in strength. Between 100 °C and 140 °C, the covalent bonds between SiO_2_, Laponite, and polymer molecular chains are less affected by temperature, so the tensile strength of the gel does not change much.

As shown in Figure 7, under different temperature conditions, the compressive strength of the hybrid crosslinked composite gel exhibits a trend of initially increasing slowly with strain, followed by accelerated growth until fracture. In the temperature range of 40–60 °C, the compressive strength remains essentially stable. When the temperature rises to 60–100 °C, the compressive strength decreases rapidly with a marked increase in deformation. In the range of 100–140 °C, the decrease in compressive strength is relatively small. This variation pattern is closely related to the thermal stability of the internal crosslinked structure within the gel [28,29]. In the range of 40–60 °C, the hydrogen bonding interactions between the Laponite nanoplatelets and polymer chains remain stable, endowing the gel with good structural integrity. When the temperature increases to 60–100 °C, these hydrogen bonds are gradually disrupted, intensifying molecular chain motion, which leads to a reduction in pressure-bearing capacity and increased deformation. In the range of 100–140 °C, although the hydrogen bonds have largely become ineffective, the covalent bond network formed between the SiO_2_ (generated from MPTMS hydrolysis) and the polymer matrix remains stable, effectively maintaining the spatial skeleton structure of the gel. Consequently, the compressive strength undergoes only minor changes. In summary, the gel relies primarily on hydrogen bonds for structural support in the low-temperature region (40–60 °C), whereas in the high-temperature region (100–140 °C), the covalent bond network dominates its mechanical performance. This temperature-dependent behavior is consistent with recent findings on the dual-crosslinking mechanism in polyacrylamide-based hydrogels, which demonstrate that covalent crosslinking plays a dominant role in structural strength under high-temperature conditions [30].

### 2.3. Lost Circulation Control Performance of the Hybrid Crosslinked Composite Gel

**Figure 8:**
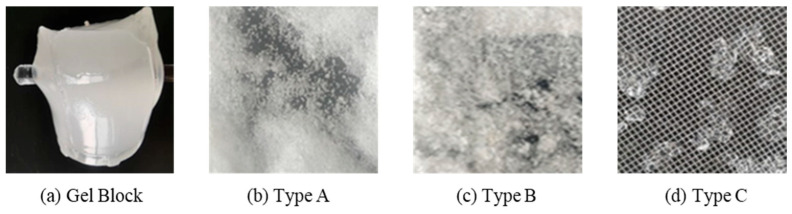
Physical images of different types of hybrid crosslinked composite gel particles.

To meet the requirements of lost circulation control technology during drilling, the hybrid crosslinked composite gel was crushed and prepared into dry gel particles of different sizes. The gel particles were dispersed and swollen in a water-based solution and then labeled as different types according to their mesh sizes. The 40–60-mesh gel particles were labeled as Type A, the 20–40-mesh gel particles were labeled as Type B, and the 10–20-mesh gel particles were labeled as Type C, as shown in Figure 8. According to the principle that the mass concentration of the three particle sizes of gel particles accounts for one-third each, different combinations of gel particles were added to a water-based drilling fluid slurry containing 4% bentonite. The resulting 1000 mL drilling fluid for lost circulation control was aged by rolling at 140 °C for 16 h. The lost circulation control performance of the hybrid crosslinked composite gel particles in porous media and fractures was then tested using a high-temperature, high-pressure dynamic and static plugging apparatus.

**Figure 9:**
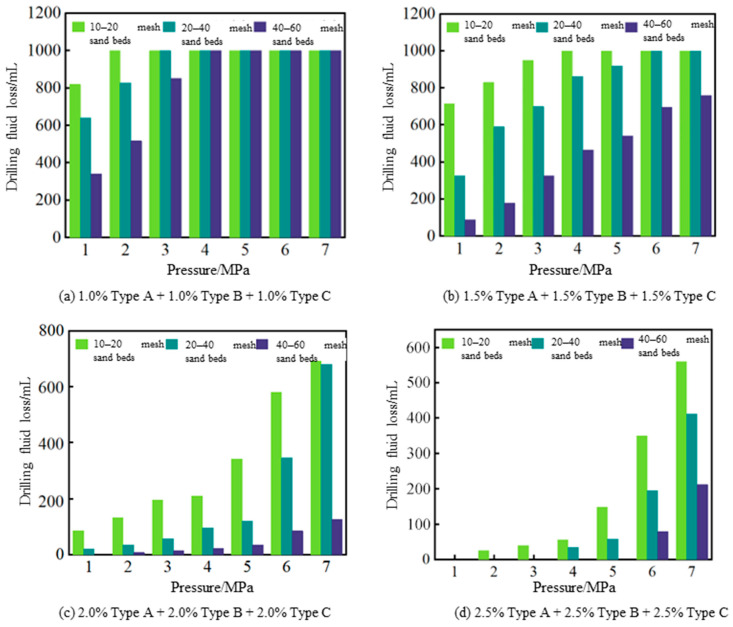
Results of sand bed sealing tests using different combinations of hybrid crosslinked gel particles.

The plugging performance of the hybrid composite gel particles in sand beds of different mesh sizes is shown in Figure 9. The experimental results indicate that when the combination of hybrid crosslinked gel particle lost circulation materials is 1.0% Type A + 1.0% Type B + 1.0% Type C (Figure 9a), the lost circulation control performance is poorest for the 10–20-mesh sand bed. At a pressure of 1 MPa, the drilling fluid loss reaches 820 mL, and complete loss occurs at 2 MPa. For the 20–40-mesh sand bed, complete loss occurs at 3 MPa. For the 40–60-mesh sand bed, the low-pressure lost circulation control performance is weak, with a loss volume of 342 mL at 1 MPa, and complete loss occurs at 4 MPa. When the combination of hybrid crosslinked gel particle lost circulation material is 1.5% Type A + 1.5% Type B + 1.5% Type C (see Figure 9b), the pressure-bearing plugging effect on sand beds with different mesh sizes is enhanced, especially for the 40–60-mesh sand bed, which does not experience complete loss even at 7 MPa. When the concentration combinations of different mesh hybrid crosslinked gel particles are 2.0% and 2.5%, respectively (see Figure 9c,d), the relationship curves between drilling fluid loss and applied pressure show a trend of initially slow increase followed by a rapid rise. Compared with the low concentration, the drilling fluid loss is significantly reduced. The data in Figure 9c show that the 40–60-mesh sand bed has a loss of only 15 mL at a pressure of 3 MPa. The data in Figure 9c show that the 20–40-mesh sand bed has a loss of only 35 mL at a pressure of 4 MPa, and the 40–60-mesh sand bed does not leak at a pressure of 5 MPa but has a loss of 79 mL at a pressure of 6 MPa. The above experimental results indicate that when the concentration combination of hybrid crosslinked composite gel particles reaches 2.0% Type A + 2.0% Type B + 2.0% Type C or higher, it can meet the requirements of lost circulation control in high-permeability formations.

**Figure 10:**
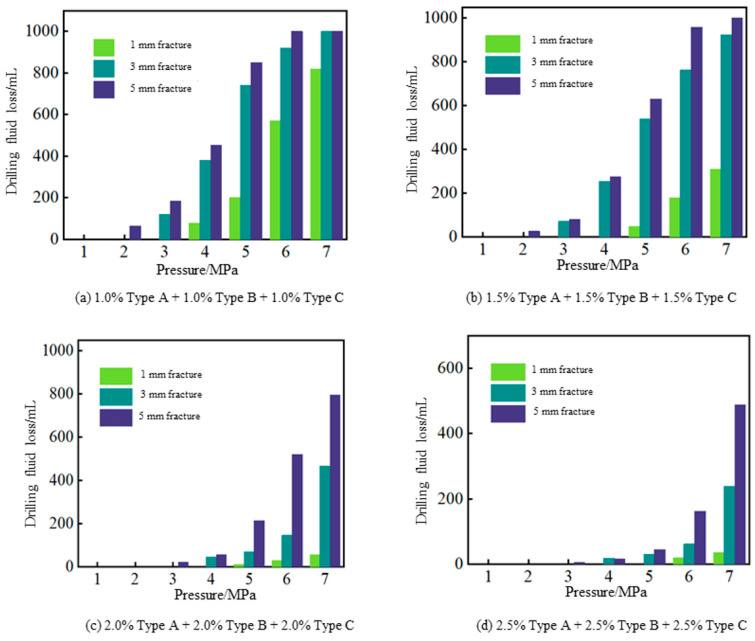
Results of fracture sealing tests using different combinations of the hybrid crosslinked gel particles.

The lost circulation control performance of hybrid crosslinked composite gel particles on fractures of different widths is shown in Figure 10. When the concentration combination of hybrid crosslinked gel lost circulation materials with different mesh sizes is 1.0%, no loss occurs at 3 MPa pressure after plugging 1 mm wide fractures, and no loss occurs at 2 MPa and 1 MPa after plugging 3 mm and 5 mm wide fractures, respectively. When the concentration combination increases to 1.5%, the pressure-bearing plugging capacity for fractures improves: for 1 mm wide fractures, no loss occurs at 4 MPa, with only 49 mL loss at 5 MPa; for 3 mm wide fractures, no loss occurs at 2 MPa; and for 5 mm wide fractures, no loss occurs at 1 MPa, with only 26 mL loss at 2 MPa. When the combination of lost circulation materials is 2.0% Type A + 2.0% Type B + 2.0% Type C, the pressure-bearing plugging capacity for fractures significantly improves: for 1 mm wide fractures, no loss occurs at 4 MPa, with only 10 mL loss at 5 MPa; for 3 mm fractures, no loss occurs at 3 MPa with 45 mL loss at 4 MPa; and for 5 mm fractures, the loss is 20 mL at 3 MPa. When the concentration combination of different mesh sizes increases to 2.5%, the pressure-bearing plugging capacity for 1 mm fractures can reach 5 MPa, with only 18 mL loss at 4 MPa for 3 mm fractures and only 5 mL loss at 3 MPa for 5 mm fractures. The above experimental results indicate that when the concentration combination of hybrid crosslinked composite gel particles reaches 2.0% Type A + 2.0% Type B + 2.0% Type C or higher, it can meet the requirements for lost circulation control in fractured formations. In field drilling operations, the concentration of 2.0% hybrid gel particles corresponds to approximately 7.0 lb/bbl for a water-based drilling fluid with a density of 1.2 g/cm^3^. Based on current raw material prices, the incremental material cost for this treatment is estimated to be 10–12 USD per barrel, which is within the typical range of high-temperature lost circulation materials currently available. While this represents an additional cost, it is justified by the reduced non-productive time and lower risk of severe lost circulation events in deep, high-temperature formations.

The above results confirm that the hybrid crosslinked composite gel particles combine the desirable viscoelasticity of monomer-polymerized gels, the high-temperature resistance of SiO_2_-reinforced gels, and the superior mechanical properties of Laponite-reinforced gels. The high elasticity of the gel is key to its plugging performance. When transported into loss channels, the gel particles resist excessive deformation, allowing them to form stable bridges at constrictions rather than being forced through fractures. This rigidity-assisted bridging mechanism, enabled by a high elastic modulus, promotes rapid accumulation and compaction of subsequent particles, resulting in a sealing layer with greater pressure-bearing capacity than conventional gels with lower elastic moduli.

### 2.4. Lost Circulation Control Mechanism of the Hybrid Crosslinked Composite Gel

**Figure 11:**
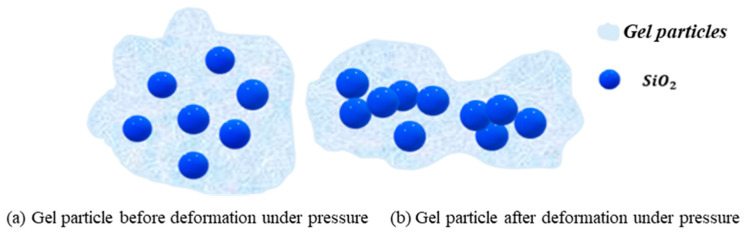
Schematic diagram of the rigid accumulation effect of SiO_2_ within hybrid crosslinked composite gel particles before and after deformation under pressure.

**Figure 12:**
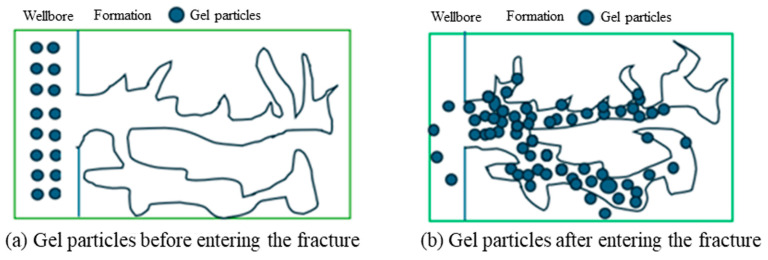
Schematic diagram of the lost circulation control mechanism of the hybrid crosslinked composite gel particles.

The lost circulation control mechanism of the hybrid crosslinked composite gel particles is similar for both porous media and fractures. Based on the rheological studies, the hybrid crosslinked composite gel particles possess excellent flexibility and can deform under pressure. Compared with rigid particles, the flexible gel particles can enter smaller lost circulation pathways and continue to deform and migrate under differential pressure, thus providing a wider matching range for the size of the loss zone [31]. Compared with pure gel particles, the lost circulation material particles in this study feature a unique “core–shell” structure. The proposed core–shell mechanism is supported by multiple lines of indirect experimental evidence. First, TEM images reveal the presence of dispersed core structures within the gel particles, which are identified as SiO_2_ nanoparticle aggregates covalently bonded to the polymer network. These aggregates act as rigid internal domains that limit overall particle deformation under pressure. Second, rheological measurements show that the gel exhibits a predominantly elastic response (G′ > G″) across the entire frequency range, with a storage modulus exceeding 3000 Pa even after aging at 140 °C. This high elastic modulus implies that the particles resist excessive deformation under differential pressure, corroborating the presence of a rigid internal framework. Third, plugging performance tests demonstrate that the hybrid gel particles achieve a pressure-bearing capacity of up to 5 MPa for 5 mm fractures, which is higher than that of conventional gel systems reported in the literature under comparable conditions [14,15]. This core–shell structure reduces the deformation range of the particles under differential pressure due to the rigid accumulation of internal SiO_2_ (Figure 11). Consequently, under the same particle size and other conditions, the particles are more prone to forming bridges at constrictions during migration within loss channels, followed by aggregation, accumulation, and compaction to fill the loss space (Figure 12). This effectively seals the loss channels and enhances the pressure-bearing capacity of the formation.

## 3. Conclusions

(1) A triple hybrid crosslinked structure was successfully constructed using MPTMS, Laponite nanoplatelets, and the reactive microgel BWL, resulting in a composite gel material with excellent high-temperature stability and mechanical properties. After aging at 140 °C, the gel maintains a high storage modulus (>4000 Pa) and loss modulus (>1000 Pa), meeting the fundamental rheological requirements for high-temperature formation plugging applications.
(2) The hybrid crosslinked structure significantly enhances the mechanical performance of the gel. Within the range of 40–60 °C, the tensile and compressive strength values of the gel exhibit minimal variation. Between 60 and 100 °C, some reduction in strength occurs due to the weakening of hydrogen bonds. In the high-temperature range of 100–140 °C, the gel retains favorable structural integrity and load-bearing capacity, owing to the covalent network formed by SiO_2_ and Laponite, demonstrating good thermal and pressure resistance stability.
(3) The hybrid gel was processed into particle-based plugging agents of varying sizes (40–60 mesh, 20–40 mesh, 10–20 mesh). The effective sealing of loss channels with different dimensions was achieved through combined usage. Experiments indicate that when the total concentration of the blended system reaches 2.0% or higher, the plugging agent system can form stable pressure-bearing seals for highly permeable sand beds and fractures with apertures up to 5 mm, with pressure-bearing capacities reaching 4–6 MPa, meeting the requirements for lost circulation control in deep, high-temperature fractured formations.

The gel material exhibits excellent high-temperature stability, mechanical strength, and plugging performance, demonstrating significant potential for lost circulation operations in deep and high-temperature fractured formations.

## 4. Materials and Methods

### 4.1. Materials and Instruments

Materials included acrylamide (AM, purity > 99.0%, Shanghai Macklin Biochemical Technology Co., Ltd., Shanghai, China); Laponite RD (industrial grade, purity 99.9%, BYK Additives (Shanghai) Co., Ltd., Shanghai, China); α-methacrylic acid (MAA, purity > 99.0%, Shanghai Macklin Biochemical Technology Co., Ltd., Shanghai, China); γ-Methacryloxypropyl trimethoxysilane (MPTMS, purity > 99.0%, Jiangsu Chenguang Coupling Agent Co., Ltd., Zhenjiang, China); reactive microgel crosslinking agent (BWL, purity > 98%, self-synthesized in laboratory); potassium persulfate (KPS, purity 99.5%, Shanghai Macklin Biochemical Technology Co., Ltd., Shanghai, China); sodium chloride (NaCl); calcium chloride (CaCl_2_); and deionized water.

Instruments included a HAAKE MARS III high-end rheometer (Thermo Fisher, Waltham, MA, USA); CMT4000 electronic universal testing machine (Shenzhen SANS Materials Testing Co., Ltd., Shenzhen, China); JSM-7900F ultra-high resolution thermal field-emission scanning electron microscope (JEOL Ltd., Tokyo, Japan); LDK high-temperature and high-pressure static/dynamic lost circulation tester (Qingdao Tongchun Petroleum Instruments Co., Ltd., Qingdao, China); and high-temperature and high-pressure physical simulation apparatus for fracture plugging (Jiangsu Haian Petroleum Technology Co., Ltd., Nantong, China).

### 4.2. Preparation of the Hybrid Crosslinked Composite Gel

Based on the total mass of the prepared gel solution, the hybrid crosslinked composite gel was synthesized as follows: Laponite at a mass concentration of 0.8% was added to deionized water and stirred thoroughly to obtain a homogeneous Laponite dispersion. Acrylamide (AM) at a mass concentration of 20% was added to the Laponite dispersion and mixed uniformly to form a Laponite/AM dispersion. MPTMS at a mass concentration of 4% and methacrylic acid (MAA) at 10% were sequentially added to the above dispersion under continuous stirring to ensure homogeneity. A reactive microgel (denoted as BWL) at a mass concentration of 0.5% was then incorporated into the mixture and stirred until uniformly dispersed. Potassium persulfate (KPS) as an initiator was added at a mass concentration of 0.1%, and the mixture was stirred thoroughly to yield the hybrid crosslinked composite gel precursor solution. The solution was purged with nitrogen gas for 20 min to remove dissolved oxygen. The degassed solution was transferred into a sealed aging vessel and allowed to react at 60 °C for 48 h, forming the hybrid crosslinked composite gel. After the reaction, the resulting gel was removed from the vessel and, depending on the intended application, was crushed and dried to produce particles of different sizes, yielding the final particulate hybrid crosslinked composite gel lost circulation material.

### 4.3. Test Methods

(1) Microstructural observation: Gel samples were first frozen in liquid nitrogen and then vacuum freeze-dried using a lyophilizer. The dried samples were brittle-fractured and sputter-coated with gold, and their microstructures were observed using an ultra-high-resolution field-emission scanning electron microscope.
(2) Rheological properties: A HAKKE Mars60 rheometer (Thermo Fisher, Waltham, MA, USA) was used to perform frequency sweep tests. The shear strain was fixed at 0.5% (within the linear viscoelastic region), and G′ and G″ were measured over an angular frequency range of 0.1–100 rad/s (approximately 0.016–16 Hz).
(3) Tensile resistance properties: The gel was molded into slender bars measuring 7 mm in length, 6 mm in width, and 6 mm in height. Tensile mechanical properties were measured using a WAW-600F universal testing machine (Jinan Xinshijin Testing Machine Co., Ltd., Jinan, China) at a crosshead speed of 100 mm/min. The stress–strain curves were recorded during the tensile process.
(4) Compression resistance properties: The gel was molded into cylindrical specimens with a base diameter of 20 mm and a height of 5 mm. Compressive mechanical properties were measured using a WAW-600F universal testing machine (Jinan Xinshijin Testing Machine Co., Ltd., Jinan, China) at a crosshead speed of 3 mm/min. The stress–strain curves were recorded during the compression process.
(5) High-temperature/high-pressure lost circulation control performance: A specified mass concentration of the lost circulation material was added to a drilling fluid base slurry (4% sodium bentonite). After full swelling, a high-temperature, high-pressure dynamic–static plugging apparatus was used to evaluate fluid loss under different applied pressures in sand beds and fracture modules of various sizes. The pressure-bearing plugging capacity in porous media and fractures was assessed accordingly. The quartz sand beds had mesh sizes of 10–20, 20–40, and 40–60, with a packed thickness of 15 cm. The measured permeabilities were approximately 12.5 D, 5.8 D, and 2.3 D, with corresponding porosities of 38%, 35%, and 32%, respectively. The fracture modules featured a single wedge-shaped fracture with inlet widths of 1 mm, 3 mm, and 5 mm, outlet widths of 0.5 mm, 1.5 mm, and 2.5 mm, and a fracture length of 15 cm.

All rheological, tensile, and compression tests were performed in triplicate (*n* = 3), and the results are reported as mean values. Due to the complexity of the experimental setup, plugging tests using sand beds and fracture modules were conducted in duplicate (*n* = 2), and the reported fluid loss volumes represent the average values.

## Data Availability

Data are contained within the article.
